# On the role of some ARGONAUTE proteins in meiosis and DNA repair in *Arabidopsis thaliana*

**DOI:** 10.3389/fpls.2014.00177

**Published:** 2014-05-20

**Authors:** Cecilia Oliver, Juan L. Santos, Mónica Pradillo

**Affiliations:** Departamento de Genética, Facultad de Biología, Universidad Complutense de MadridMadrid, Spain

**Keywords:** ARGONAUTE proteins, small RNAs, meiosis, DNA repair, *Arabidopsis*

## Abstract

In plants, small non-coding RNAs (≈20–30 nt) play a major role in a gene regulation mechanism that controls development, maintains heterochromatin and defends against viruses. However, their possible role in cell division (mitosis and meiosis) still remains to be ascertained. ARGONAUTE (AGO) proteins are key players in the different small RNA (sRNA) pathways. *Arabidopsis* contains 10 AGO proteins belonging to three distinct phylogenetic clades based on amino acid sequence, namely: AGO1/AGO5/AGO10, AGO2/AGO3/AGO7, and AGO4/AGO6/AGO8/AGO9. To gain new insights into the role of AGO proteins, we have focused our attention on AGO2, AGO5, and AGO9 by means of the analysis of plants carrying mutations in the corresponding genes. AGO2 plays a role in the natural cis-antisense (nat-siRNA) pathway and is required for an efficient DNA repair. On the other hand, AGO5, involved in miRNA (microRNA)-directed target cleavage, and AGO9, involved in RNA-directed DNA methylation (RdDM), are highly enriched in germline. On these grounds, we have analyzed the effects of these proteins on the meiotic process and also on DNA repair. It was confirmed that AGO2 is involved in DNA repair. In *ago2-1* the mean cell chiasma frequency in pollen mother cells (PMCs) was increased relative to the wild-type (WT). *ago5-4* showed a delay in germination time and a slight decrease in fertility, however the meiotic process and chiasma levels were normal. Meiosis in PMCs of *ago9-1* was characterized by a high frequency of chromosome interlocks from pachytene to metaphase I, but chiasma frequency and fertility were normal. Genotoxicity assays have confirmed that AGO9 is also involved in somatic DNA repair.

## Introduction

ARGONAUTE (AGO) proteins are essential players in the different small RNA (sRNA) pathways. sRNAs are non-coding RNAs, about 20–30 nt in length, involved in regulating gene expression. Silencing/repression occurs by transcriptional gene silencing (TGS), by recruitment of histone and/or DNA methyltransferases to regulatory sequences of target genes, or by post-transcriptional gene silencing (PTGS), blocking either the proper translation or cleaving the target mRNA (Wassenegger et al., 1994; Xie et al., 2004). There are three classes of sRNAs: microRNAs (miRNAs), which are genome-encoded and derived from imperfectly folded stem-loop structures of single stranded RNA precursors (ssRNAs); small interfering RNAs (siRNAs), which derive from long double-stranded RNA precursors (dsRNAs); and PIWI-interacting RNAs (piRNAs) which are animal germline specific (Castel and Martienssen, 2013). In *Arabidopsis* there are three classes of siRNAs: repeated associated siRNAs (ra-siRNAs), involved in RNA-directed DNA methylation pathway (RdDM) and acting at TGS level; natural antisense siRNAs (nat-siRNAs), derived from natural antisense transcription and involved in PTGS; and trans-acting siRNAs (ta-siRNAs) which act at PTGS level. These sRNAs are firstly processed from longer RNA precursors by a member of the DICER RNase III like endonuclease family, that cleaves the long dsRNAs to generate small dsRNAs about 20–30 nt (Matzke and Birchler, 2005). One strand of this dsRNAs associates with silencing effector complexes through AGO proteins. 5′ nucleotide identity, together with sRNA length, contribute to the sorting of sRNAs into these AGO proteins (Mi et al., 2008; Takeda et al., 2008; Havecker et al., 2010).

AGO proteins were named after the observation of a phenotype in an *Arabidopsis* knockout mutant (*ago1*) whose leaves closely resembled the tentacles of a small squid (Bohmert et al., 1998). They show a great variability in the number and diversity among organisms, from a single AGO protein in fission yeast to 10 and 19 in *Arabidopsis* and rice, respectively (Wood et al., 2002; Kapoor et al., 2008; Vaucheret, 2008). In *Arabidopsis*, three distinct clades based on amino acid sequence homology have been identified, namely: AGO1/AGO5/AGO10 (involved in miRNA pathway), AGO2/AGO3/AGO7 (involved in nat-siRNA and ta-siRNA pathways), and AGO4/AGO6/AGO8/AGO9 (involved in ra-siRNA pathway) (Vaucheret, 2008). These sequence-based clades concur with the three functional groups established. Thus, members of the AGO1/5/10 clade are RNA slicers, AGO2/3/7 bind sRNAs (AGO7 also cuts), and AGO4/6/8/9 are chromatin modifiers (Montgomery et al., 2008; Havecker et al., 2010; Ji et al., 2011). Within each clade there is a main player with ubiquitous and high level expression (AGO1, AGO7, and AGO4), while other members are flower/embryo-specific players (AGO5/10, AGO2/3, and AGO8/9) (Schmid et al., 2005; Takeda et al., 2008; Mallory et al., 2009). Transcriptomic analyses have shown that *AGO2, AGO5*, and *AGO9* are predominantly expressed in ovules (Olmedo-Monfil et al., 2010; Wuest et al., 2010). AGO5 promotes the transition to megagametogenesis in the functional megaspore (Tucker et al., 2012). By contrast, AGO9 and 24 nt siRNAs prevent that sub-epidermal cells can adopt a megaspore-like identity, being crucial to specify cell fate in the ovule (Olmedo-Monfil et al., 2010). Thus, AGO9 restricts reproductive potential to the functional megaspore and AGO5 promotes the initiation of megagametogenesis in this cell (Tucker et al., 2012).

With respect to male germ cells, there are different classes of miRNAs and ta-siRNAs with more enriched expression in mature pollen grains than in leaves (Borges et al., 2008, 2011; Takeda et al., 2008; Grant-Downton et al., 2009). Also, *AGO9* and other genes related with RdDM are expressed in pollen grains (Borges et al., 2008). On the other hand, *AGO2*, *AGO5*, and *AGO9* are expressed in both uninuclear and binuclear pollen grains. *AGO5* and *AGO9* are also expressed in trinuclear and mature pollen grains (Grant-Downton et al., 2009). A possible role of sRNAs during plant meiosis is only exemplified by rice *MEIOSIS ARRESTED AT LEPTOTENE1* (*MEL1*, related to *Arabidopsis AGO5*), and maize *AGO104* (related to *Arabidopsis AGO9)*. The former regulates cell division of premeiotic germ cells and the faithful progression of meiosis, but not their initiation and establishment (Nonomura et al., 2007). The latter represses somatic fate in germ cells. Hence, *ago104* pollen mother cells (PMCs) show alterations in chromatin condensation, spindle formation, and chromosome segregation (Singh et al., 2011). On the other hand, Wei et al. (2012) have reported that AGO2 is involved in DNA double-strand break (DSB) repair by homologous recombination (HR). In this context, it must be taken into account that meiotic programmed DSBs are essential for HR and the correct segregation of homologous chromosomes at meiosis. Aiming to contribute to a better understanding of the possible role of AGO2 (At1g31280), AGO5 (At2g27880), and AGO9 (At5g21150) in both male meiosis and DNA repair, we have analyzed the phenotypes displayed by the corresponding mutant plants in relation to these processes. We have also included *ago3* and *ago8* mutants in the analysis because *AGO3* (At1g31290) and *AGO8* (At5g21030) are recent duplications of *AGO2* and *AGO9*, respectively.

## Materials and methods

### Plant materials and growth conditions

All T-DNA insertion mutants analyzed belong to the Columbia accession (Col). Seeds were obtained from the Salk Institute Genomic Analysis Laboratory (SiGnAL, http://signal.salk.edu/cgi-bin/tdnaexpress; Alonso et al., 2003), and provided by the Nottingham Arabidopsis Stock Centre (NASC). The mutants studied were *ago2-1* (SALK_003380), *ago3-2* (SALK_005335), *ago5-4* (SALK_050483), *ago8-1* (SALK_139894), and *ago9-1* (SALK_127358). Plants were cultivated on a soil mixture of vermiculite and commercial soil (3:1) and grown in a greenhouse under a 16-h light/8-h dark photoperiod, at 18–20°C with 70% humidity. Genotyping of each mutant was performed by PCR using a combination of three primers, one T-DNA specific primer, LBb1.3 (5′-ATTTTGCCGATTTCGGAAC-3′), and two specific primers for the corresponding gene: LP*AGO2* (5′-CCATTGTAGGGCTGAGTATGC-3′) and RP*AGO2* (5′-CGTTTCCCTGTGGCCTGAACA-3′); LP*AGO3* (5′-CGATAGTCCCGACTGACTCTG-3′) and RP*AGO3* (5′-AAACAGAGAGACAGTGGACGC-3′); LP*AGO5* (5′-CTACCCATCAGGGAGCTAAGG-3′) and RP*AGO5* (5′-TTTCTGGACCATATCACAGCC-3′); LP*AGO8* (5′-TCCCTGTTTTGGTTCCTTTTC-3′) and RP*AGO8* (5′-TCCTGTTCCTGTTTCCATGAC-3′); LP*AGO9* (5′-GGGATACATCCATCCCAACA-3′) and RP*AGO9* (5′-AGGCCGTATCTTACCACACC-3′).

### Cytological analysis

Fixation of flower buds, slide preparations of PMCs, and fluorescence *in situ* hybridization (FISH) were carried out according to Sánchez-Morán et al. (2001). The DNA probes used comprise ribosomal DNA 5S and 45S loci to identify individual bivalents (Gerlach and Bedbrook, 1979; Campell et al., 1992), and a 180 bp repeat sequence (pAL1) to detect centromeres (Martínez-Zapater et al., 1986). These probes were labeled with either biotin-dUTP or digoxygenin-dUTP, using a nick translation kit (Roche). For 5-methylcytosine immunodetection, the slide preparations were dried at 60°C (30 min), fixed in 1% formaldehyde (10 min), dehydrated through an ethanol series (70, 90, 100%) and then dried at room temperature. After denaturation using HB50 at 80°C (2 min), slides were rinsed in ice-cold 2×SSC and incubated in 1% BSA (1 h). The preparations were then washed three times in PBS and one in TNT (5 min each). For the immunodetection, slides were incubated with anti-5-methylcytosine raised in mouse (1:50, Eurogenetec) during 30 min and washed three times in TNT (5 min). Finally, a FITC-conjugated anti-mouse antibody (1:500, Abcam) was used.

### DNA damage sensitivity assays

To test the sensitivity to γ-rays, sterilized seeds were kept in sterile water at 4°C for approximately 24 h and then were exposed to 100, 200, 300, 400, and 500 Gy doses (2.94 Gy/min) from a ^137^Cs source (IBL 437C; CISBIO Bioassays). After irradiation seeds were germinated on MS agar medium. For mitomycin C (MMC, Duchefa) assay, 4-day-old seedlings were transferred to liquid GM with different MMC concentrations (3, 6, 9, and 12 μg/ml). For cisplatin [cis-diamminedichloroplatinum (II), CDDP, Sigma] assay, seeds were germinated on MS agar medium with different CDDP concentrations (15, 30, 50, and 75 μ M). The effects of the individual DNA damage agents on plant growth were evaluated 12 days (MMC) or 14 days (γ-rays, CDDP) after sowing.

## Results

We confirmed previous results in our growing conditions, homozygous *ago2-1* and *ago9-1* plants being indistinguishable from wild-type (WT) plants with respect to vegetative growth (Havecker et al., 2010; Harvey et al., 2011). Rosette leaves appeared normal and bolting was not delayed. Also, the length of siliques and the number of seeds per silique were similar to those of WT, hence, they were fully fertile. However, homozygous *ago5-4* plants showed a delay in germination time and a slight decrease in fertility.

### Meiotic observations in PMCs

**Figure 1:**
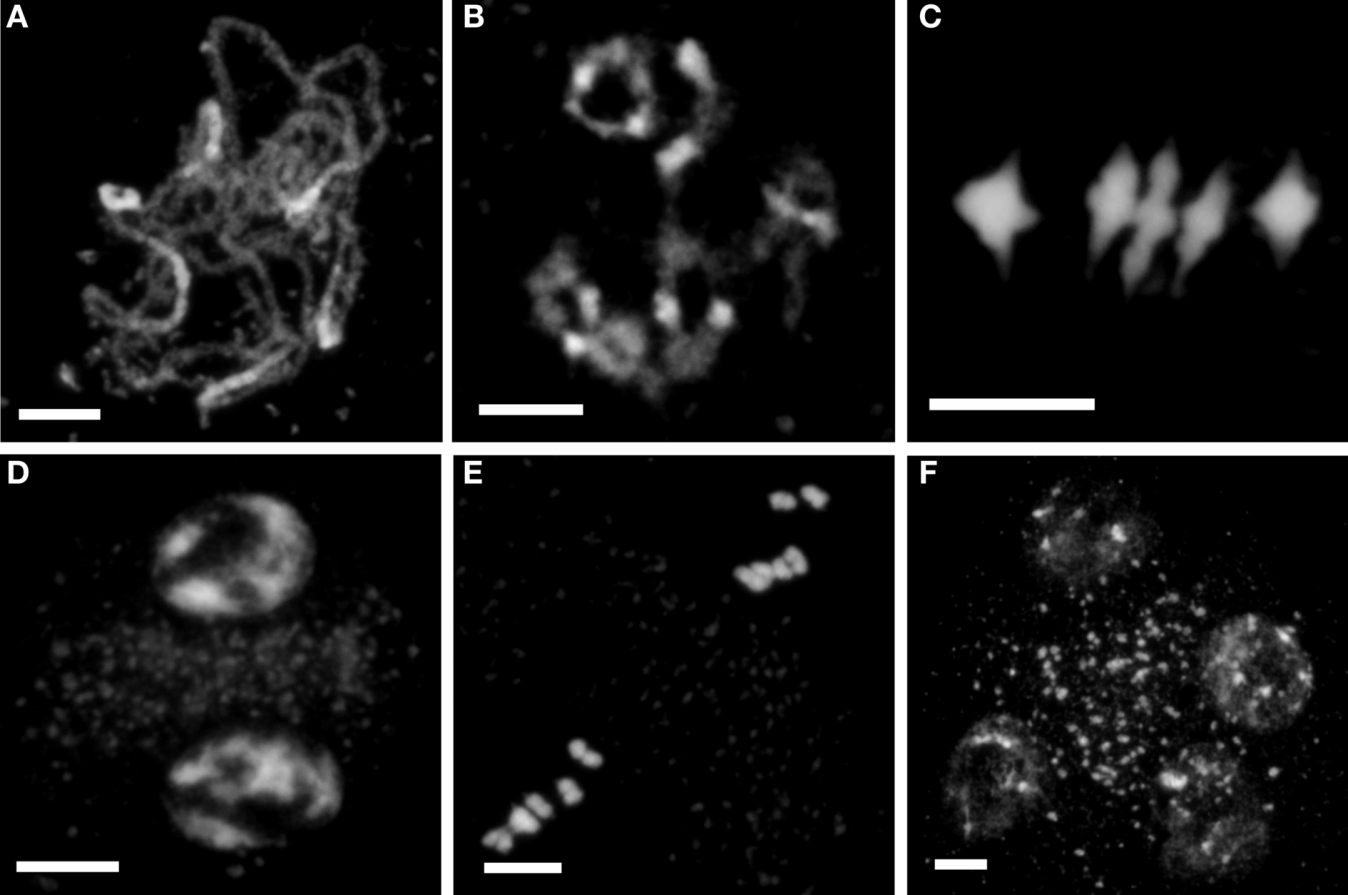
**Meiosis is apparently normal in PMCs of *ago2-1*, *ago5-4* and *ago9-1***. No significant meiotic abnormalities were observed in PMCs of the mutants *ago2-1*, *ago5-4* and *ago9-1*, excluding the interlocks displayed by *ago9-1* (see text and Figure 2 for more details). Meiotic progression in *ago5-4* is shown as example. **(A)** Fully synapsed pachytene. **(B)** Diakinesis. **(C)** Metaphase I in which the five bivalents are properly aligned. **(D)** Prophase II. Five chromosomes are distinguished on opposite sides of the organelle band. **(E)** Metaphase II. Condensation progresses normally and two sets of five aligned chromosomes are observed. **(F)** Tetrad with four haploid cells that contain five chromatids each. Bars represent 5 μm.

We compared different stages of meiotic progression after 4′,6-diamidino-2-phenylindole (DAPI) staining of PMCs from WT and mutant plants. Our observations indicate that in all mutants the crucial processes of pairing, synapsis and HR at first meiotic division occur normally, and also the second division until tetrad formation (Figure 1).

**Figure 2:**
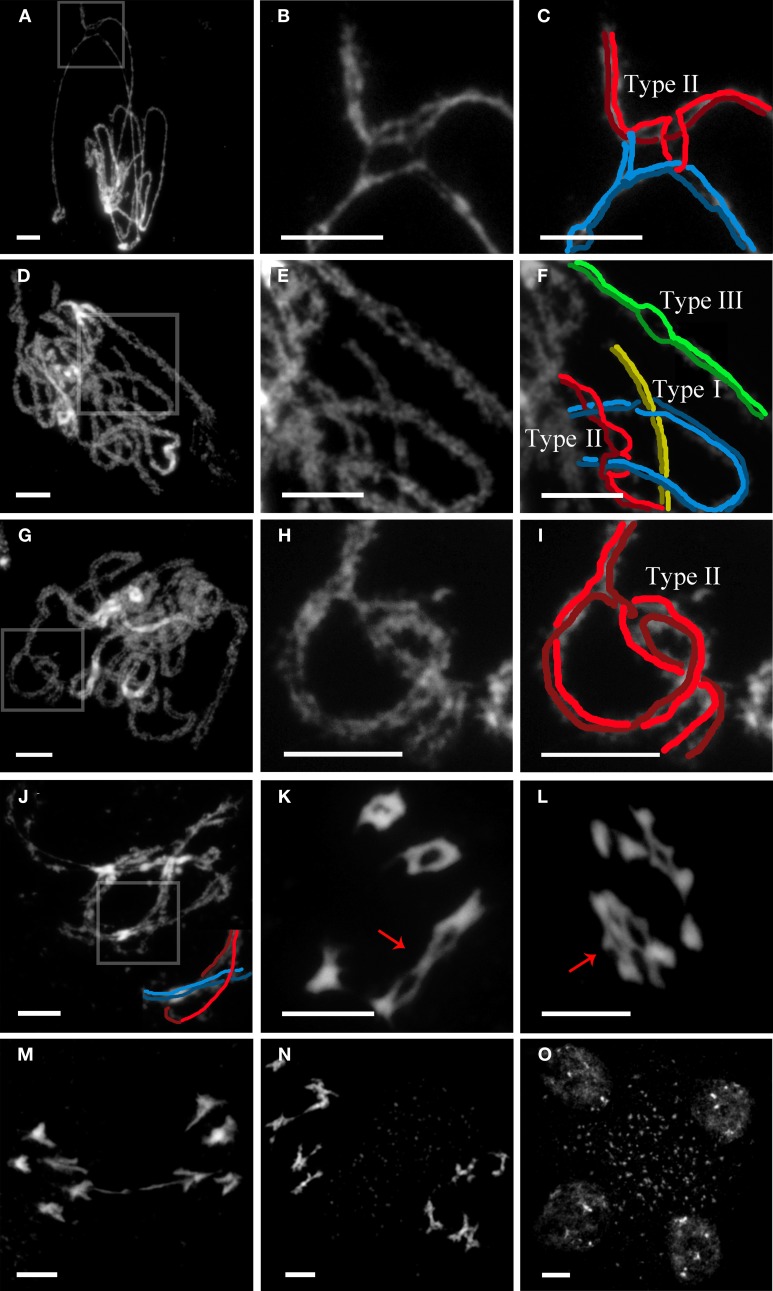
***ago9-1* displays interlocks (ILs) during late prophase I that are successfully resolved**. **(A,D,G)** Examples of *ago9-1* pachytenes. **(B,E,H)** Details of the ILs from **(A,D,G)**, respectively. **(C,F,I)** Possible interpretations of the ILs from **(A,D,G)**, respectively. **(J)** Diplotene with a type I IL. A schematic representation of the IL is also provided. **(K)** Diakinesis with two trapped bivalents (arrow). **(L)** Early anaphase I in which two bivalents appear joined by an IL (arrow). **(M)** Anaphase I showing a delay in chromatid separation, perhaps as consequence of the existence of previous ILs. **(N)** Prometaphase II with five chromosomes in opposite sides of the organelle band. **(O)** Normal tetrad. Bars represent 5 μm.

In WT meiosis, unrelated chromosomes are occasionally entangled with one another, usually at zygotene. This fact leads to the production of interlocks (ILs). In such situations, either a bivalent (type I IL) or one unsynapsed chromosome (type II IL) can be trapped between two aligned homologous chromosomes, held in place by regular synapsis to either side (Wang et al., 2009). Thus, ILs are always associated with unsynapsed regions, indicating that they can prevent or hinder normal synaptonemal complex (SC) formation. In *Arabidopsis*, observations of surface spread meiocytes under electron microscope revealed a mean value of around one IL per nucleus (in cells with more than 70% of synapsis) (López et al., 2008). Later on, by mid-late pachytene, no ILs were observed. Thus, WT meiosis must include a mechanism for their resolution during zygotene-pachytene. In the absence of AGO9, and in striking contrast to WT, 40% of the mid-late pachytene nuclei (*n* = 43) showed at least one IL (Figures 2A–I). In addition, we have also observed a new IL configuration (type III), only previously reported in *Sordaria* (Storlazzi et al., 2010), where two homologous chromosomes show nonsynapsed regions (variable in length) not involved in ILs (Figure 2F). These authors have suggested that these configurations could represent cases of incomplete resolution and/or regions that are unable to synapse after IL resolution. It is likely that this high frequency of ILs in *ago9-1* can be responsible for a delay in the duration of meiosis at prophase I since the number of diplotene nuclei observed was substantially higher than in WT. Even we observed ILs during this stage (Figure 2J) and onwards (Figures 2K,L). The occurrence of bridges at anaphase I could be consequence of difficulties in IL resolution (Figure 2M). However, the second division was completely normal in *ago9-1* PMCs (Figures 2N,O).

**Figure 3:**
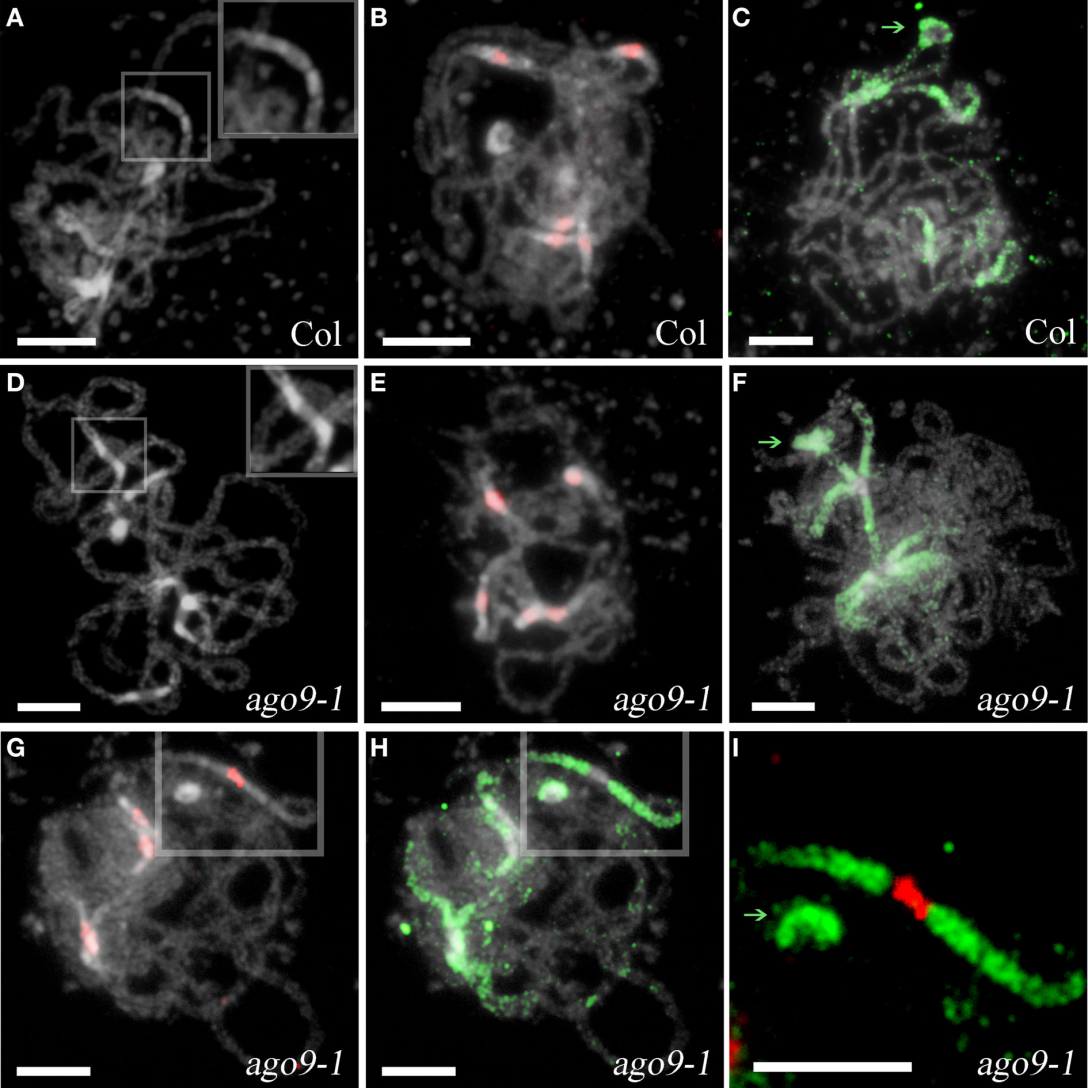
**Analysis of heterochromatic pericentromeric regions at pachytene in *ago9-1***. **(A,D)** Pericentromeric regions are distinctly defined at Col and *ago9-1* pachytenes. A detail of these regions is showed at the right top of each picture. **(B,E)** Detection of centromeric regions (using pAL1 as probe, red). **(C,F)** Detection of pericentromeric regions (5-methylcytosine, green). **(G–I)** Simultaneous location of centromeric and pericentromeric regions does not reveal differences between Col and *ago9-1* plants. A detail of one bivalent is shown in **(I)**. NORs are indicate by green arrows. Bars represent 5 μm.

Maize *AGO104*, an ortholog of *Arabidopsis AGO9*, appears to be required for heterochromatic CHG and CHH methylation (Singh et al., 2011). Also, Havecker et al. (2010) have reported the involvement of AGO9 in the siRNA-directed maintenance of the silencing state of several classes of repetitive DNA, and Durán-Figueroa and Vielle-Calzada (2010) have pointed out that AGO9 is necessary in the ovule to inactivate transposable elements located in the pericentromeric regions of all five chromosomes of *Arabidopsis.* Taking into account these results and, since centromeric and pericentromeric regions of *ago9-1* chromosomes appeared slightly different after DAPI staining respect to WT (Figures 3A,D), we decided to look for changes in 5-methylcytosine distribution, since this modification is mostly associated with heterochromatic regions in plants (Mathieu et al., 2002). No differences between the mutant and WT were found (Figures 3C,F–I). Likewise, no changes in the size and intensity of a signal corresponding to a centromeric probe were observed (Figures 3B,E,G–I).

### Chiasma analysis

Chiasma frequency estimations in *Arabidopsis* are best cytologically assessed at metaphase I where, despite the disadvantage of maximum chromosome condensation there is no risk of confusing chiasmata and relational twists. On the other hand, condensation represents an advantage when FISH is applied. The chromosomes of *Arabidopsis* can be individually distinguished for the purpose of chiasma counting following FISH with probes for 45S and 5S rDNA (Sánchez-Morán et al., 2001, 2002; López et al., 2012).

**Figure 4:**
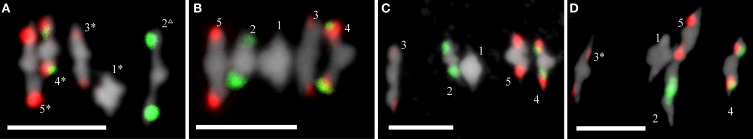
**FISH images of metaphase I cells from wild-type, *ago2-1*, *ago5-4*, and *ago9-1* plants**. **(A)** Col. **(B)** *ago2-1*. **(C)** *ago5-4*. **(D)** *ago9-1*. 45S and 5S rDNA loci are identified by green and red signals, respectively. Bivalents are numbered in each cell. As example, four ring bivalents (marked by an asterisk) and a rod bivalent (triangle) are observed in **(A)**. Bars represent 5 μm.

Chiasma frequencies were recorded from 41 to 69 FISH-labeled metaphase I PMCs per WT and each of the mutants, according to the criteria established previously by Sánchez-Morán et al. (2001). Data were collected from two or three plants per genotype. Since there were no significant differences in the mean cell chiasma frequencies per cell between them, individual plant data were grouped. In all of the analyzed cells, the five chromosome pairs always form five bivalents, either rods or rings (Figure 4). Rods are bound by chiasmata in one arm, whereas rings have both arms bound by chiasmata. The mean chiasma frequencies per cell, per bivalent, and per bivalent arm are summarized in Table 1 and Supplementary Table S1. *ago2-1* showed a significant increase of the mean cell chiasma frequency per cell respect to WT, mainly due to an excess of chiasmata in the long arm of chromosome 4. In contrast, no significant differences for this parameter were observed between WT and *ago3-2, ago5-4*, *ago8-1*, and *ago9-1*. However, except in *ago3-2*, there was an increase in the chiasma frequency of the short arm of chromosome 4 in all of these mutants.

**Table 1:** **Mean chiasma frequencies per cell, per bivalent and per bivalent arm (short vs. long) in the Col accession, *ago2-1, ago5-4*, and *ago9-1***.

**Bivalents**											***C***	***n***
	**1**		**2**		**3**		**4**		**5**			
	**s**	**l**	**s**	**l**	**s**	**l**	**s**	**l**	**s**	**l**		
Col	–	–	0.61	1.14	0.90	1.26	0.48	1.01	0.97	1.30	10.20	69
	2.52 (0.25)		1.75 (0.17)		2.16 (0.21)		1.49 (0.15)		2.28 (0.22)			
*ago2-1*	–	–	0.62	1.16	0.96	1.13	0.65	1.29^**^	1.00	1.36	10.73^*^	55
	2.55 (0.24)		1.78 (0.17)		2.09 (0.19)		1.95^***^ (0.18)		2.36 (0.22)			
*ago5-4*	–	–	0.67	0.86^***^	1.00^**^	1.02^***^	0.76^**^	1.02	1.00	1.19	10.05	42
	2.52 (0.25)		1.53^*^ (0.15)		2.02 (0.20)		1.78^**^ (0.18)		2.19 (0.22)			
*ago9-1*	–	–	0.88^**^	1.02^*^	0.92	1.12	0.76^**^	1.07	0.98	1.29	10.46	41
	2.41 (0.23)		1.90 (0.18)		2.05 (0.20)		1.83^**^ (0.17)		2.27 (0.22)			

C, mean chiasma frequencies per cell; n, number of cells; s, short arm; l, long arm; Asterisks indicate P-values from t-Student tests:

*** P < 0.001,

** P < 0.01, and

* P < 0.05

### DNA damage sensitivity assays

**Figure 5:**
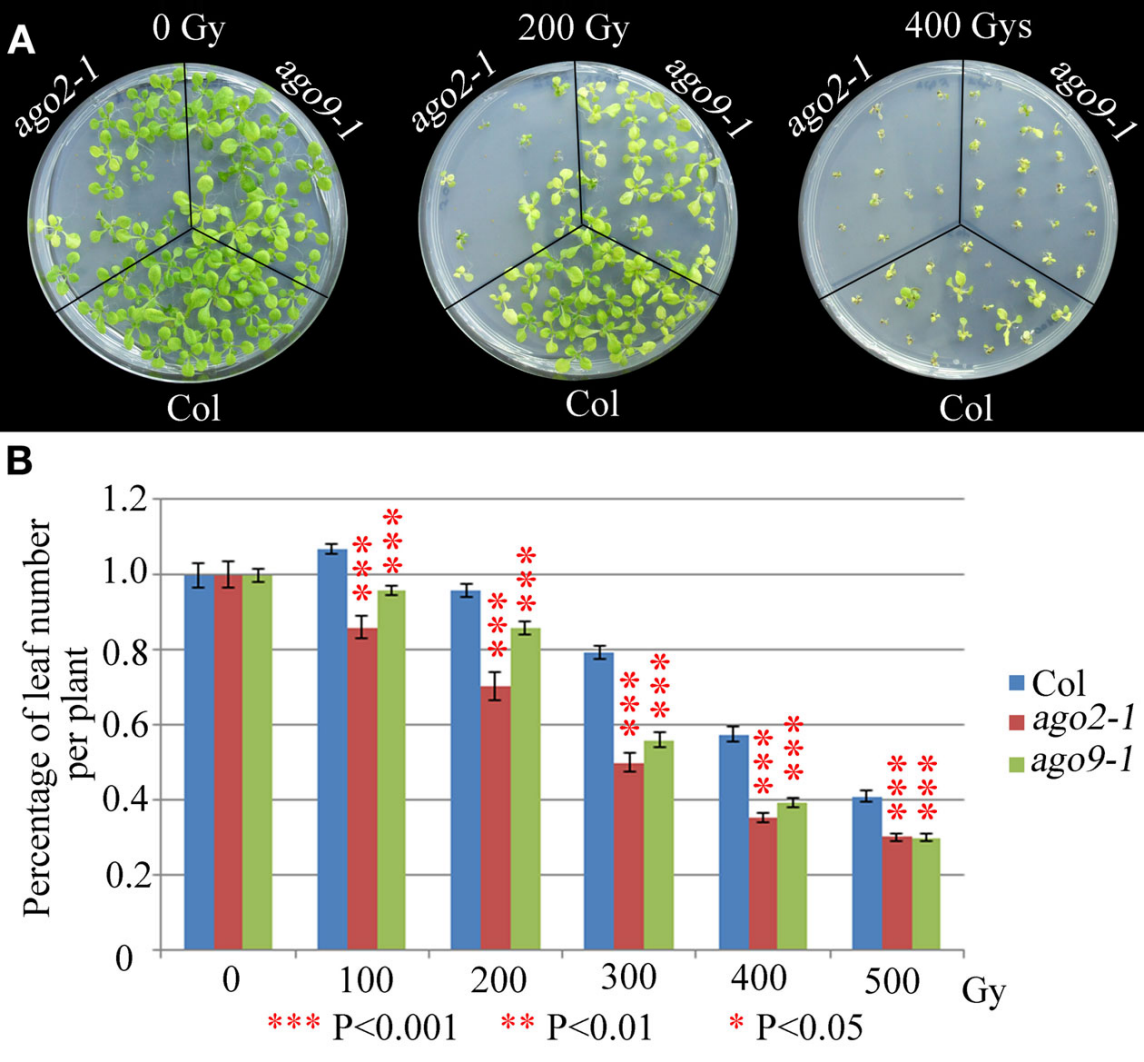
***ago2-1* and *ago9-1* are hypersensitive to γ-rays. (A)** Phenotypes of 14-day-old seedlings (*ago2-1, ago9-1*, and Col) after treatment with different radiation doses. **(B)** Percentage of leaf number per plant after treatment with different radiation doses. Leaf number at each dose was scored and put into relation to the leaf number of the untreated plantlets of the same line. Mean values and standard errors are depicted. Asterisks indicate *P*-values from *t*-Student tests: ^***^P < 0.001, ^**^P < 0.01, and ^*^P < 0.05. See Supplementary Table S2 for more details.

**Figure 6:**
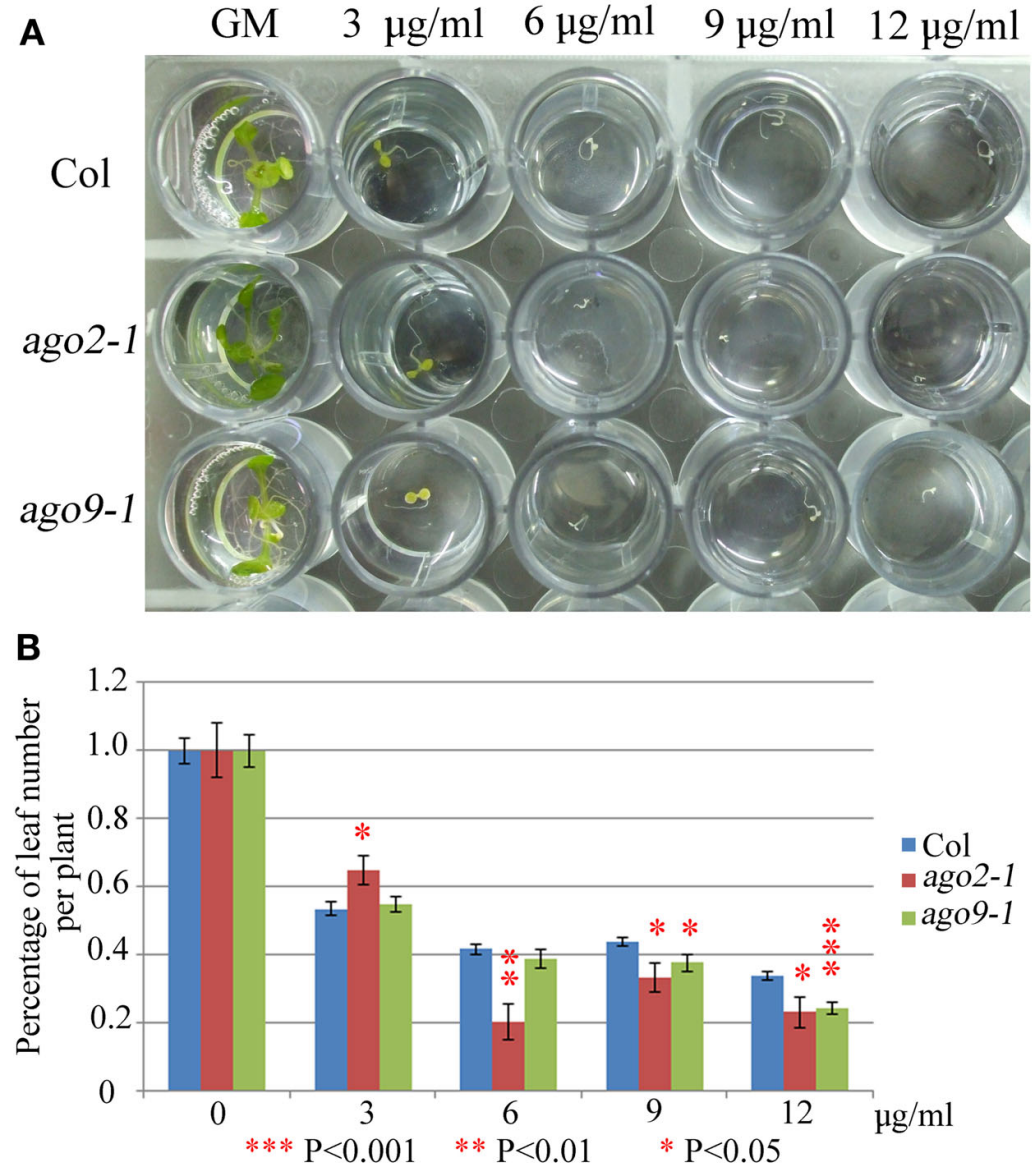
***ago2-1* and *ago9-1* are hypersensitive to MMC. (A)** Phenotypes of 16-day-old seedlings (*ago2-1, ago9-1*, and Col) grown on liquid media containing different concentrations of MMC. **(B)** Percentage of leaf number per plant after treatment with different concentrations. Leaf number at each dose was scored and put into relation to the leaf number of the untreated plantlets of the same line. Mean values and standard errors are depicted. Asterisks indicate *P*-values from *t*-Student tests: ^***^P < 0.001, ^**^P < 0.01, and ^*^P < 0.05. See Supplementary Table S2 for more details.

**Figure 7:**
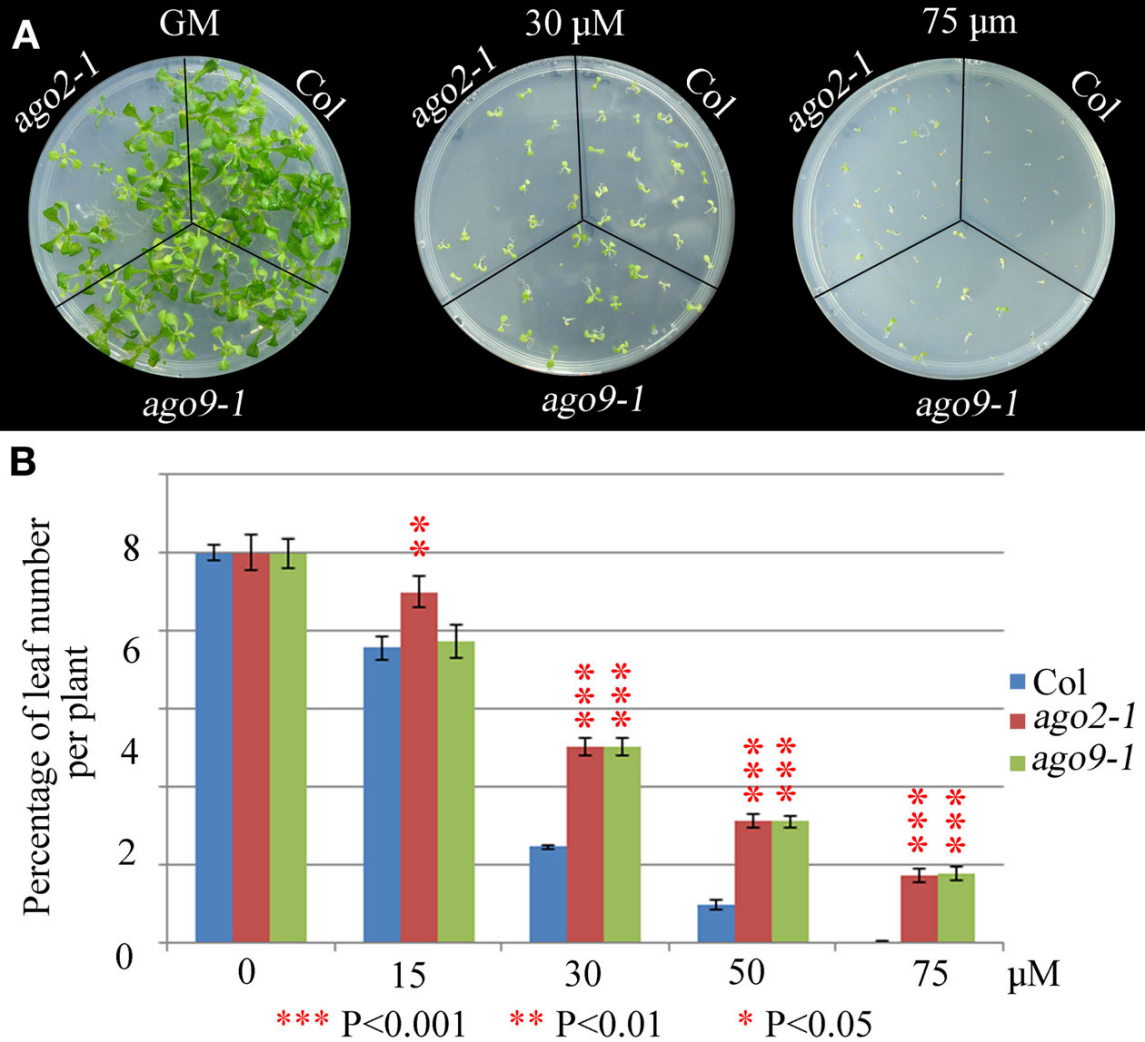
**WT plants are more sensitive to CDDP than *ago2-1* and *ago9-1* plants. (A)** Phenotypes of 14-day-old seedlings (*ago2-1, ago9-1*, and Col) sown on media containing different concentrations of CDDP. **(B)** Percentage of leaf number per plant after treatment with different concentrations. Leaf number at each dose was scored and put into relation to the leaf number of the untreated plantlets of the same line. Mean values and standard errors are depicted. Asterisks indicate *P*-values from *t*-Student tests: ^***^P < 0.001, ^**^P < 0.01, and ^*^P < 0.05. See Supplementary Table S2 for more details.

We have explored whether AGO2 and AGO9 could play a global role in DNA repair by using γ-irradiation and the cross-linking (CL) agents MMC and CDDP. Unfortunately, we were unable to perform this kind of experiments in *ago5-4* due to its delay in germination time. We verified that *ago2-1* and *ago9-1* plants are hypersensitive to γ-rays because after a 100 Gy dose and beyond, they showed a reduction in the percentage of leaf number per plant with respect to WT (Figure 5, Supplementary Table S2), which was also accompanied with a parallel decrease in the fresh weight per plant (Supplementary Table S2). The results obtained also indicated that *ago2-1* is more sensitive to γ-irradiation than *ago9-1.* Further, *ago2-1* and *ago9-1* plants are hypersensitive to MMC and, again, *ago2-1* resulted to be more sensitive to this reagent than *ago9-1.* In the former the decrease of the parameters analyzed took place at a concentration of 6 μg/ml, while in the latter was at 9 μg/ml (Figure 6, Supplementary Table S2). By contrast, both *ago* mutants are not hypersensitive to CDDP, even they turned to be more resistant to CDDP than WT from 30 μM concentration and beyond (Figure 7, Supplementary Table S2).

## Discussion

### ago2-1

AGO2 localizes to both the nucleus and the cytoplasm (Takeda et al., 2008). Although several publications have reported the involvement of this protein in the viral siRNA pathway (Blevins et al., 2006; Harvey et al., 2011; Wang et al., 2011), its implication in the nat-siRNA pathway is not completely understood. nat-siRNA are originated by the co-expression of overlapping sense/antisense transcripts which triggers the formation of dsRNAs. Once processed into 24-nt nat-siRNAs, they are probably loaded onto AGO2 (and/or AGO3) to conduct the cleavage of homolog transcripts, producing 21-nt-nat-siRNAs (Jin et al., 2008; Jamalkandi and Masoudi-Nejad, 2009). This kind of endogenous siRNAs have been related to environmental stress responses and to the sperm function control during double fertilization (Borsani et al., 2005; Ron et al., 2010; Yao et al., 2010). Further, Wei et al. (2012) detected the production of sRNAs, named diRNAs (DSB-induced sRNAs), in both plant and human cells, generated by DNA damage from the sequences around DSB sites. In *Arabidopsis*, diRNAs are recruited by AGO2 to mediate DSB repair and their production is dependent on the protein kinase ATR, which has an essential role during DNA damage response (Culligan and Britt, 2008). Wei et al. (2012) also reported that diRNAs probably function downstream of ATM/ATR-dependent H2AX phosphorylation, one of the earliest events after DSB formation (Amiard et al., 2010). Previously, Culligan et al. (2006) demonstrated that *AGO2* can be induced by γ-irradiation and Wei et al. (2012) confirmed that the expression of *AGO2*, but not the expression other *AGOs*, was highly induced in plants upon γ-irradiation at both mRNA and protein levels. Our results corroborate the role of AGO2 in DSB repair because *ago2-1* plants not only are hypersensitive to γ-rays (Figure 5) but also to MMC (Figure 6). However, we obtained opposite results by using CDDP because *ago2-1* plants were more resistant to this agent than WT plants (Figure 7).

While γ-irradiation is a powerful DSB-inducing agent, MMC mainly produces inter-strand CLs on DNA (Rink et al., 1996) and CDDP preferentially forms intra-strand CLs (Eastman, 1985). Both types of CLs induce DSBs during DNA synthesis which are mostly repaired by HR. However, some DNA polymerases can bypass intra-strand CLs, making this type of lesion less toxic than that produced by inter-strand CLs. In G1 cells nucleotide excision repair (NER) can remove a subset of CLs (Deans and West, 2011). The results obtained here indicate that AGO2 (and diRNAs) is likely more involved in pathways for reparing inter-strand CLs than in those for repairing intra-strand CLs.The opposite situation was observed in *Arabidopsis recq4* plants (defective for the helicase RECQ4) which showed high sensitivity for CDDP but no sensitivity against to MMC (Mannuss et al., 2010), reinforcing the idea that both agents induce at least partially different kinds of DNA damage. In this sense a large proportion of the genes induced by γ-irradiation and CL agents overlaps, but there is a specific subset of genes induced by each alone (Chen et al., 2003; West et al., 2004; Culligan et al., 2006). On the other hand, the resistant phenotype of *ago2-1* in response to CDDP might be explained by the fact that HR blocking (and hence the generation of toxic intermediates) could improve the efficiency of other DNA repair pathways, in this case related to the elimination of intra-strand CLs. Indeed, a recent publication has demonstrated that in mammals, AGO2 forms a complex with the recombinase RAD51, indispensable during HR, and promotes its recruitment to DSB sites (Gao et al., 2014).

In *Arabidopsis*, meiotic HR, initiated by programmed DSBs, is required for proper synapsis of homologous chromosomes and meiotic progression. Hence, and taking into account the role of AGO2 in DSB repair efficiency, we decided to analyze the chromosome behavior in PMCs of *ago2-1* plants. At cytological level, meiosis appeared normal (Figures 1, 4) with the only mention that mean cell chiasma frequency was higher than WT (Table 1). Defects in HR and increases in chiasma frequency have also been reported in plants with mutations in *FANCM* (Fanconi anemia complementation group M), which encodes for a DNA translocase required for the repair of DNA inter-strand CLs (Crismani et al., 2012; Knoll et al., 2012). This finding is particularly interesting *per se*, but it is also worth of mention that chromosome 4, especially its long arm, is the only responsible for the increase in chiasma frequency. In this context, it has also been reported that a particular chromosome may differ in chiasma frequency among either different mutants or accessions (Sánchez-Morán et al., 2001; Perrella et al., 2010; López et al., 2012).

AGO2 and AGO3 are included in the same *Arabidopsis* clade. The corresponding genes are very similar to each other, probably they arose from a recent duplication event and are adjacent to one another in the genome, suggesting the two proteins have similar activities and/or redundant functions (Baumberger and Baulcombe, 2005; Vaucheret, 2008). However, since the mean cell chiasma frequency of *ago3-2* was similar to that displayed by the WT (Supplementary Table S1), it is tempting to speculate that the functions of both proteins, at least on meiotic HR, could be different.

### ago5-4

*AGO5*, a close paralog of *AGO1*, directs sRNA-mediated gene expression regulation for all currently characterized *Arabidopsis* miRNAs (Vaucheret et al., 2004; Baumberger and Baulcombe, 2005). However, in contrast to *AGO1*, *AGO5* expression profile is highly specific to reproductive tissues (Schmid et al., 2005) and accumulates in sperm cell cytoplasm, mature pollen, and growing pollen tubes (Borges et al., 2011). It is also expressed around reproductive cells during megagametogenesis, *ago5-4* plants being defective in the initiation of this process (Tucker et al., 2012). The rice genome contains at least five paralogs (*MEL1* group) of *Arabidopsis AGO5* (Kapoor et al., 2008), but only one exhibits an expression profile similar to *AGO5*, *MEL1*. In the seed-sterile *mel1-1* mutant, PMCs are arrested at early meiosis I (only ~5% of PMCs escaped meiotic arrest) and meiotic chromosomes present condensation defects, although axial elements are formed (Nonomura et al., 2007). These authors have proposed that MEL1 is involved in the organization of nucleolar organizing regions (NORs) because in the mutant the chromosome axis protein PAIR2 associates with the nucleolus, a situation never observed in WT cells.

Despite the decrease in fertility observed in homozygous *ago5-4* plants, meiosis in PMCs was cytologically normal (Figures 1, 4). Although no differences in chiasma frequency respect to WT were observed (Table 1), NOR-bearing chromosomes (2 and 4), and also chromosome 3 displayed a significant increase in the number of chiasmata.

### ago9-1

*AGO9* is a close paralog of *AGO4* and is involved in the ra-siRNA pathway, participating in the maintenance of the silencing state of several classes of repetitive DNA element (Havecker et al., 2010). Like *AGO2* and *AGO3*, *AGO8* and *AGO9* are almost adjacent each other, suggesting they have arisen from a recent gene duplication event, although *AGO8* is probably a pseudogene (Takeda et al., 2008; Vaucheret, 2008). Initially characterized by the absence of any obvious developmental defect (Havecker et al., 2010), a closer examination of *ago9* mutant revealed the differentiation of multiple gametic cells from somatic companion cells that are able to initiate gametogenesis (Olmedo-Monfil et al., 2010). However, any publication has analyzed a possible role of AGO9 (and ra-siRNAs) in DNA damage repair. The results obtained were quite similar to those reported for *ago2*. Thus, *ago9-1* was hypersensitive to both γ-irradiation and MMC, although in minor extent that *ago2-1*, and also was more resistant against CDDP than WT (Figures 5–7; Supplementary Table S1), revealing a possible role for ra-siRNAs and RdDM in the repair or inter-strand CLs and DSBs.

Mutations in maize *AGO104* (related to *AGO9*) produced unreduced female gametes generating an apomixes-like fertilization-independent seed production phenotype. Also, the absence of AGO104 generate severe defects during male meiosis: abnormal condensation during diakinesis-metaphase I, absence of a bipolar spindle at anaphase I and dyads with partial chromosome condensation and irregular spindle formation, which lead to the formation of triads and microspores with multiple nuclei (Singh et al., 2011). However, our results have revealed normal meiosis in PMCs of *ago9-1* (Figures 1, 4) with the exception of the high frequency of ILs observed at pachytene, some of them maintained until metaphase I (Figure 2). Since ILs have been observed in many organisms, the exceeding low rate of meiosis failure in WT meiocytes implicates the existence of specific mechanisms to deal with them (Zickler and Kleckner, 1999). One model proposed for their resolution suggests movement of the interlocked chromosomes out through the ends of the constraining bivalents (Scherthan et al., 2007; Conrad et al., 2008; Koszul et al., 2008). However, we have not observed apparent differences on centromere and telomere dynamics at prophase I between *ago9-1* and WT PMCs. Another model invokes DNA topoisomerase II in axis breakage and reformation (Von Wettstein et al., 1984). In addition, Storlazzi et al. (2010) have found that MLH1, a central player in HR, is required for IL resolution, suggesting that this process requires elimination of constraining DNA connections formed by the recombination process. Thus, the hypersensitivity to γ-rays and MMC could be related with a defect in HR, revealing an involvement of AGO9 in this process, that could also be the reason of the ILs observed. Further studies will be necessary to comfirm this hypothesis.

AGO9 is important for heterochromatic silencing (Havecker et al., 2010). Even, Durán-Figueroa and Vielle-Calzada (2010) have provided evidence that AGO9 predominantly targets transposable element in the pericentromeric regions of all five chromosomes in the ovule. Also, AGO104 has a strong effect on centromeric repeats and controls non-CG DNA methylation at centromeric heterochromatin (Singh et al., 2011). Although these facts could be related with the different chromatin conformation observed in pericentromeric regions of *ago9-1* chromosomes respect to the WT, this is not apparently the case (Figure 3). Additional experiments at molecular level will be required to determine the reason of these observations.

Finally, although mean cell chiasma frequency in *ago9-1* plants was not different to that of WT (Table 1), chiasma formation was again significantly increased in short arms of NOR-bearing chromosomes 2 and 4. The same also occurs in *ago8-1* (Supplementary Table S1).

Summing up, the results obtained in this work have confirmed that AGO2 has a role in DNA repair, but also that AGO9 could be involved in this process. The fact that both mutants are hypersensitive to both γ-irradiation and MMC, and resistant to CDDP respect to WT constitutes an exciting result because it opens a door to the possibility that these proteins may be involved in a specific pathway of DNA repair, related to the resolution of inter-strand CLs (and/or DSBs). On the other hand, the increase of chiasma frequency, especially in NOR-bearing chromosomes, observed in the analyzed mutants is intringuing and merits further investigation. Finally, it is important to mention that the absence of a clear meiotic phenotype in these mutants could be due to overlapping functions of AGO proteins. The analysis of double or triple mutants, in the case they were viable, could contribute to obtain more information about the function of these proteins.

### Conflict of interest statement

The authors declare that the research was conducted in the absence of any commercial or financial relationships that could be construed as a potential conflict of interest.
