# Supplementary material for: On the role of some ARGONAUTE proteins in meiosis and DNA repair in Arabidopsis thaliana
Source: Front Plant Sci. 2014 May 20;5:177. doi: 10.3389/fpls.2014.00177 (PMC4033075; doi:10.3389/fpls.2014.00177)
Supplement: Supplementary file 1 [file DataSheet1.DOCX]

***Supplementary Material***

**On the role of some ARGONAUTE proteins in meiosis and DNA repair in *Arabidopsis thaliana***

**Cecilia Oliver*, Juan L Santos and Mónica Pradillo**

Departamento de Genética, Facultad de Biología, Universidad Complutense de Madrid, Madrid, Spain

***Correspondence:** Cecilia Oliver, Departamento de Genética, Facultad de Biología, Universidad Complutense de Madrid, Madrid, Spain

ceoliver@ucm.es

**Supplementary Tables**

**Supplementary Table S1.** Mean chiasma frequencies per cell, per bivalent and per bivalent arm (short *vs.* long) in *ago3-2* and *ago8-1*.

|  | Bivalents | | | | | | | | | | | | | |  |  |
| --- | --- | --- | --- | --- | --- | --- | --- | --- | --- | --- | --- | --- | --- | --- | --- | --- |
|  | 1 | | 2 | | | 3 | | | | 4 | | | 5 | |  |  |
|  | s | l | | s | l | | s | l | s | | l | s | | l | C | n |
| *ago3-2* | - | - | | 0.66 | 1.14 | | 0.91 | 1.20 | 0.64 | | 1.02 | 0.98 | | 1.25 | 10.27 | 44 |
|  | 2.48 (0.24) | | | 1.80 (0.18) | | | 2.11 (0.21) | | 1.67 (0.16) | | | 2.22 (0.22) | | |  |  |
| *ago8-1* | - | - | | 0.85** | 1.15 | | 0.97 | 1.10 | 0.80*** | | 1.10 | 0.98 | | 1.28 | 10.55 | 61 |
|  | 2.33* (0.22) | | | 2.00* (0.19) | | | 2.07 (0.20) | | 1.90*** (0.18) | | | 2.26 (0.21) | | |  |  |

C, mean chiasma frequencies per cell; n, number of cells; s, short arm; l, long arm;

Asterisks indicate P values from t-Student tests: ***P < 0.001, **P < 0.01 and *P < 0.05

**Supplementary Table S2.** DNA damage sensitivity assays in *ago2-1* and *ago9-1*.

| DNA damage agent |  | Dose | **Col** | ***ago2-1*** | | ***ago9-1*** | |
| --- | --- | --- | --- | --- | --- | --- | --- |
|  |  |  | Mean | Mean | t-student | Mean | t-Student |
| **Gamma rays** | Leaves per plant | 0 Gy | 6.99 ± 0.23 | 6.64 ± 0.23 | 1.03 | 7.71 ± 0.13 | -2.73** |
|  |  | 100 Gy | 7.47 ± 0.10 | 5.71 ± 0.20 | 8.09*** | 7.38 ± 0.10 | 0.68 |
|  |  | 200 Gy | 6.70 ± 0.13 | 4.67 ± 0.24 | 7.50*** | 6.61 ± 0.13 | 0.47 |
|  |  | 300 Gy | 5.55 ± 0.13 | 3.32 ± 0.16 | 10.75*** | 4.32 ± 0.15 | 6.21*** |
|  |  | 400 Gy | 4.01 ± 0.14 | 2.33 ± 0.08 | 10.12*** | 3.04 ± 0.11 | 5.39*** |
|  |  | 500 Gy | 2.86 ± 0.10 | 2.00 ± 0.08 | 7.03*** | 2.31 ± 0.07 | 4.64*** |
|  | Percentage of leaves per plant | 0 Gy | 1.00 ± 0.03 | 1.00 ± 0.03 | 0.00 | 1.00 ± 0.02 | 0.00 |
|  |  | 100 Gy | 1.07 ± 0.01 | 0.86 ± 0.03 | 6.45*** | 0.96 ± 0.01 | 5.89*** |
|  |  | 200 Gy | 0.96 ± 0.02 | 0.70 ± 0.04 | 6.34*** | 0.86 ± 0.02 | 4.00*** |
|  |  | 300 Gy | 0.79 ± 0.02 | 0.50 ± 0.02 | 9.51*** | 0.56 ± 0.02 | 8.62*** |
|  |  | 400 Gy | 0.57 ± 0.02 | 0.35 ± 0.01 | 9.26*** | 0.39 ± 0.01 | 7.18*** |
|  |  | 500 Gy | 0.41 ± 0.01 | 0.30 ± 0.01 | 6.051*** | 0.30 ± 0.01 | 6.67*** |
|  | Fresh weight per plant (mg) | 0 Gy | 8.61 | 4.18 |  | 9.06 |  |
|  |  | 100 Gy | 8.64 | 3.95 |  | 9.30 |  |
|  |  | 200 Gy | 6.61 | 2.57 |  | 5.59 |  |
|  |  | 300 Gy | 3.98 | 1.37 |  | 3.25 |  |
|  |  | 400 Gy | 2.10 | 0.70 |  | 1.22 |  |
|  |  | 500 Gy | 0.89 | 0.33 |  | 0.62 |  |
| **Mitomycin C** | Leaves per plant | 0 µg/ml | 4.31 ± 0.16 | 3.00 ± 0.24 | 4.79*** | 4.15 ± 0.19 | 0.65 |
|  |  | 3 µg/ml | 2.31 ± 0.09 | 1.94 ± 0.13 | 2.41* | 2.28 ± 0.09 | 0.20 |
|  |  | 6 µg/ml | 1.80 ± 0.07 | 0.61 ± 0.16 | 7.00*** | 1.62 ± 0.12 | 1.31 |
|  |  | 9 µg/ml | 1.89 ± 0.06 | 1.00 ± 0.13 | 6.32*** | 1.56 ± 0.10 | 2.90** |
|  |  | 12 µg/ml | 1.46 ± 0.06 | 0.69 ± 0.13 | 5.24*** | 1.02 ± 0.08 | 4.53*** |
|  | Percentage of leaves per plant | 0 µg/ml | 1.00 ± 0.04 | 1.00 ± 0.08 | 0.00 | 1.00 ± 0.05 | 0.00 |
|  |  | 3 µg/ml | 0.53 ± 0.02 | 0.65 ± 0.04 | -2.80* | 0.55 ± 0.02 | -0.50 |
|  |  | 6 µg/ml | 0.42 ± 0.02 | 0.20 ± 0.05 | 3.93*** | 0.39 ± 0.03 | 0.83 |
|  |  | 9 µg/ml | 0.44 ± 0.01 | 0.33 ± 0.04 | 2.32* | 0.38 ± 0.02 | 2.29* |
|  |  | 12 µg/ml | 0.34 ± 0.01 | 0.23 ± 0.04 | 2.30* | 0.24 ± 0.02 | 4.08*** |
|  | Fresh weight per plant (mg) | 0 µg/ml | 12.36 | 14.00 |  | 13.65 |  |
|  |  | 3 µg/ml | 1.17 | 12.86 |  | 0.96 |  |
|  |  | 6 µg/ml | 0.16 | 25.09 |  | 0.14 |  |
|  |  | 9 µg/ml | 0.26 | 7.78 |  | 0.09 |  |
|  |  | 12 µg/ml | 0.21 | 9.33 |  | 0.10 |  |
| **Cisplatin** | Leaves per plant | 0 µM | 8.36 ± 0.16 | 6.27 ± 0.28 | 6.43*** | 7.02 ± 0.26 | 4.34*** |
|  |  | 15 µM | 6.34 ± 0.25 | 5.65 ± 0.24 | 1.98 | 5.42 ± 0.30 | 2.36* |
|  |  | 30 µM | 2.07 ± 0.04 | 3.16 ± 0.15 | -7.33*** | 3.52 ± 0.15 | -9.44*** |
|  |  | 50 µM | 0.82 ± 0.09 | 1.97 ± 0.10 | -8.28*** | 2.18 ± 0.12 | -9.18*** |
|  |  | 75 µM | 0.04 ± 0.03 | 1.08 ± 0.11 | -9.21*** | 1.25 ± 0.13 | -9.28*** |
|  | Percentage of leaves per plant | 0 µM | 1.00 ± 0.02 | 1.00±0.05 | 0.00 | 1.00 ± 0.04 | 0.00 |
|  |  | 15 µM | 0.76 ± 0.03 | 0.90±0.04 | -2.93** | 0.77 ± 0.04 | -0.26 |
|  |  | 30 µM | 0.25 ± 0.00 | 0.50±0.02 | -10.92*** | 0.50 ± 0.02 | -11.68*** |
|  |  | 50 µM | 0.10 ± 0.01 | 0.31±0.02 | -10.88*** | 0.31 ± 0.02 | -11.17*** |
|  |  | 75 µM | 0.00 ±0.00 | 0.17±0.02 | -9.41*** | 0.18 ± 0.02 | -9.39*** |
|  | Fresh weight per plant (mg) | 0 µM | 28.82 | 13.85 |  | 25.29 |  |
|  |  | 15 µM | 15.30 | 11.15 |  | 15.85 |  |
|  |  | 30 µM | 2.19 | 3.78 |  | 4.02 |  |
|  |  | 50 µM | 0.72 | 1.38 |  | 1.61 |  |
|  |  | 75 µM | 0.28 | 0.46 |  | 0.60 |  |

Asterisks indicate P values from t-Student tests: ***P < 0.001, **P < 0.01 and *P < 0.05
